# EVOLUTION OF DIVERGENT FEMALE MATING PREFERENCE IN RESPONSE TO EXPERIMENTAL SEXUAL SELECTION

**DOI:** 10.1111/evo.12473

**Published:** 2014-07-21

**Authors:** Allan Debelle, Michael G Ritchie, Rhonda R Snook

**Affiliations:** 1 Animal & Plant Sciences, University of Sheffield, Alfred Denny BuildingSheffield, S10 2TN, United Kingdom; 2 School of Biology, University of St AndrewsSt Andrews, Fife, KY16 9TH, United Kingdom

**Keywords:** Coevolution, courtship song, Drosophila, experimental evolution, population divergence, speciation

## Abstract

Sexual selection is predicted to drive the coevolution of mating signals and preferences (mating traits) within populations, and could play a role in speciation if sexual isolation arises due to mating trait divergence between populations. However, few studies have demonstrated that differences in mating traits between populations result from sexual selection alone. Experimental evolution is a promising approach to directly examine the action of sexual selection on mating trait divergence among populations. We manipulated the opportunity for sexual selection (low vs. high) in populations of *Drosophila pseudoobscura*. Previous studies on these experimental populations have shown that sexual selection manipulation resulted in the divergence between sexual selection treatments of several courtship song parameters, including interpulse interval (IPI) which markedly influences male mating success. Here, we measure female preference for IPI using a playback design to test for preference divergence between the sexual selection treatments after 130 generations of experimental sexual selection. The results suggest that female preference has coevolved with male signal, in opposite directions between the sexual selection treatments, providing direct evidence of the ability of sexual selection to drive the divergent coevolution of mating traits between populations. We discuss the implications in the context sexual selection and speciation.

In theory, sexual selection has the ability to drive the coevolution of male and female mating traits (i.e., mating signals and their associated mating preferences) within populations (Fisher 1930). If such coevolution occurs in different directions among populations, this can lead to the nonoverlapping distributions of mating traits among populations, resulting in individuals from a population being unwilling to mate with individuals from another population (and vice versa), hence potentially initiating or completing a speciation event (Lande 1981; West-Eberhard 1983; Panhuis et al. 2001; Kirkpatrick and Ravigné 2002; Ritchie 2007; ITN Marie Curie Speciation 2011; Maan and Seehausen 2011). Following the first models of divergent coevolution of mating traits via runaway sexual selection (Lande 1981; Kirkpatrick 1982), multiple theoretical studies have confirmed the potential of sexual selection to drive mating trait divergence among populations through sexually antagonistic or other forms of sexual selection, eventually resulting in sexual isolation (Turner and Burrows 1995; Higashi et al. 1999; Gavrilets 2000; Kirkpatrick and Ravigné 2002; Uyeda et al. 2009). Yet, despite its theoretical potential to influence reproductive isolation, sexual selection is often thought more likely to generate mating trait divergence by acting in partnership with divergent ecological selection (Coyne and Orr 2004; Rundle and Nosil 2005; Ritchie 2007; Sobel et al. 2010; ITN Marie Curie Speciation 2011; Maan and Seehausen 2011). Several key studies have demonstrated a crucial role of ecologically based sexual selection in driving mating traits divergence and leading to speciation (Boughman 2001; Boughman et al. 2005; Maan et al. 2006). However, strong empirical support for the ability of sexual selection alone to drive mating trait divergence between populations is still largely lacking, or is only indirect (Panhuis et al. 2001; Ritchie 2007; ITN Marie Curie Speciation 2011; Maan and Seehausen 2011; Rodríguez et al. 2013; Safran et al. 2013).

For example, evidence used to support speciation via sexual selection is frequently historic, based on phylogenetic associations between species richness and the degree of mating trait differences or sexual dimorphism (reviewed in Kraaijeveld et al. 2010). This correlative approach cannot distinguish either the chronology of mating trait divergence (e.g., whether mating trait divergence was at the origin of the speciation process or whether it occurred secondarily and maintained divergence) or the nature of the evolutionary process at the origin of mating trait divergence (e.g., sexual selection, ecologically based sexual selection, sex-specific ecological selection, or reinforcement) (Ritchie 2007; Hoskin and Higgie 2010; Kraaijeveld et al. 2010). Moreover, because these meta-analyses often use male morphological traits as a proxy for the intensity of sexual selection, they cannot confirm the coevolution of female mating preference in such divergence (Ritchie 2007). Contemporary studies of wild populations can show the match between mating signals and preferences within species and their divergence across species, but still cannot address the nature of the evolutionary process that led to mating trait divergence in the first place. Some studies, however, provide strong indirect evidence that sexual selection alone contributed to the origin of reproductive isolation by driving mating trait divergence between populations. For example, explosive radiation events, such as the Hawaiian *Laupala* crickets (Shaw 2000; Mendelson and Shaw 2005), in which closely related species clearly differ in mating traits, and yet no apparent ecological divergence is observed, suggest that the divergent coevolution of mating traits was solely due to sexual selection. Artificial selection has shown direct evidence for coevolution between a given mating signal and its associated mating preference within species (Houde 1994; Wilkinson and Reillo 1994; Brooks and Couldridge 1999). Using experimental populations of a stalk-eyed fly species, Wilkinson and Reillo (1994) selected for increased and decreased male eye span, a male mating signal, for 13 generations and found a correlated response of female mating preference for male eye span. However, these artificial selection studies commonly produced asymmetric or transient responses of mating trait evolution to selection (e.g., Houde 1994; Wilkinson and Reillo 1994). Moreover, artificial selection constrains the response of phenotypes to determined phenotypic directions, which limits understanding how sexual selection may drive mating trait divergence. Thus, we still lack direct conclusive evidence of whether sexual selection can generate mating trait divergence on its own.

One way to directly address the evolution of traits under sexual selection is to employ experimental evolution. Experimental evolution represents a powerful approach for studying mating trait evolution via sexual selection, as it allows the manipulation of the opportunity for sexual selection (and hence potentially sexual selection intensity) on replicated populations across multiple generations and the observation of the evolutionary consequences of this manipulation on male and female phenotypes (Garland and Rose 2009). Two studies demonstrated female mating preference divergence as a response to sexual selection using experimental evolution in *Drosophila serrata* (Rundle and Chenoweth 2005; Rundle et al. 2009). However, these studies examined the role of sexual selection during adaptation to a novel resource environment, and therefore the action of sexual selection occurred alongside the action of ecological adaptation. These findings highlight the need for further research on the divergence of mating preference for a mating signal as the sole result of sexual selection manipulation.

In this study, we investigate the divergence of female mating preference for a male mating signal, the interpulse interval (IPI) of Drosophila courtship song, in experimentally evolved populations of *Drosophila pseudoobscura* in which the opportunity for sexual selection has been manipulated by subjecting replicate populations to either enforced monogamy or elevated polyandry. Courtship song is composed of several acoustic signals under female choice in numerous *Drosophila* species (Hoikkala et al. 1998; Ritchie et al. 1999; Saarikettu et al. 2005), and IPI is correlated with male mating success in *D. pseudoobscura*. The mating success of backcross hybrids between *D. pseudoobscura* and *D. persimilis*, its sister species with a longer IPI, is higher with *D. pseudoobscura* females when IPI is short (Williams et al. 2001). IPI has responded to sexual selection intensity as predicted in our experimentally evolved populations of *D. pseudoobscura*. IPI was consistently shorter in polyandrous (male-biased) lines than in monogamous lines after 30 generations (Snook et al. 2005) and 110 generations of selection (Debelle 2013). Here, we assess whether sexual selection has also driven the coevolution of female preference using a playback design to measure differences in female preference functions for IPI between polyandrous and monogamous populations. If female preference has coevolved with male IPI, then we expect that females from the polyandrous and monogamous lines will prefer a song similar to the song of males from their respective treatment. Female preference in Drosophila is usually scored as both mating latency and the number of matings (Ritchie et al. 1999; Rybak et al. 2002; Martin and Hosken 2003; Talyn et al. 2004; Bacigalupe et al. 2007), so we specifically predict that mating latency will be the shortest and mating more likely to occur within lines of the same sexual selection treatment than between lines of different sexual selection treatments.

## Material and Methods

### SEXUAL SELECTION TREATMENTS

The establishment and maintenance of the selection lines are described in detail in Crudgington et al. (2005). Briefly, an ancestral wild-caught population of *D. pseudoobscura* from Tucson (AZ; allopatric to *D. persimilis*), a naturally polyandrous species (wild-caught females have been shown to be frequently inseminated by at least two males at any given time; Anderson 1974), was used to establish the selection lines. From this population, four replicate lines (replicate 1, 2, 3, and 4) of two different sexual selection treatments were established. To modify the opportunity for sexual selection, adult sex ratio in vials is manipulated by either confining one female with a single male (“monogamy” treatment; M) or one female with six males (“elevated polyandry” treatment; E) in vials. Effective population sizes are equalized between the treatments (Snook et al. 2009). At each generation, offspring are collected and pooled together for each replicate line, and a random sample from this pool is used to constitute the next generation in the appropriate sex ratios, thus proportionally reflecting the differential offspring production across families. In total, eight selection lines (M1, M2, M3, M4 and E1, E2, E3, E4) are maintained, in standard vials (2.5 × 80 mm) and with a generation time of 28 days. The ancestral population (A) is maintained in bottles (57 × 132 mm) with an equal sex ratio of adult flies. All populations are kept at 22°C on a 12L:12D cycle, with standard food media and added live yeast.

### EXPERIMENTAL FLIES

Both A, M, and E females were used to test for female mating preference coevolution with male IPI. To generate experimental females, parents were collected from each line, at the following generations: replicate 1 = 135; replicate 2 = 134; replicate 3 = 133; replicate 4 = 131; ancestral population = 155. We standardized for maternal and larval environments as previously described (Crudgington et al. 2010), but, in brief, parents were mated en masse in food bottles, transferred to bottles with oviposition plates (Snook and Markow 2001), allowed to oviposit for 24 h, and then 48 h later, 100 first instar larvae were seeded in standard food vials. Virgin E, M, or A females were collected on the day of eclosion and kept in vials of 12 individuals for five days, to ensure reproductive maturity (Snook and Markow 2001).

We used playback (see section Playback design) to measure female mating preference functions (e.g., Ritchie 1996; Shaw 2000; Shaw and Herlihy 2000). Because *Drosophila* courtship song is produced by wing vibration, males used in playback experiments must be prevented from singing. This is usually achieved through the complete ablation of both wings (Ritchie et al. 1999; Rybak et al. 2002; Talyn et al. 2004). However, preliminary playback experiments using wingless *D. pseudoobscura* males resulted in very few matings (five out of 300 pairs tested; A. Debelle, unpubl. data). Matings were restored when wings were left partially intact. Partial ablation was performed by clipping both wings using a micro-scalpel under CO_2_ anesthetization longitudinally along a segment from the posterior part of the wing base to a point on the anterior wing margin located between the ends L2 and L3 veins (see Fig. S1), therefore drastically reducing the wing area and consequently the ability of a male to sing (Ewing 1964; Sivinski and Webb 1985). We only used virgin males from the ancestral (A) population so that any residual male song would be consistent across all trials. Virgin males were wing clipped at three days old, then kept in vials of 12 individuals for a further two days, to be used in playback trials.

### ARTIFICIAL SONG CONSTRUCTION

**Figure 1:**
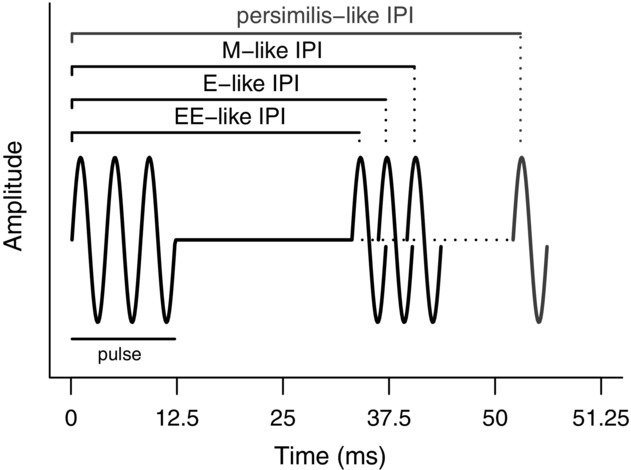
Representation of the four artificial courtship songs synthesized. The figure represents the interval of time between two consecutive pulses of song (IPI) of the four songs. All song parameters values are given in Table1. To test for an effect of a further exaggeration of E-like IPI (an even shorter IPI), the difference between E-like and M-like IPI was subtracted to E-like IPI value, to create an EE-like IPI song. M is for monogamous, and E is for polyandrous.

To measure female mating preference functions for male courtship song in each population, four artificial courtship songs with different IPI values were synthesized. These were played back to females of each population, and the female mating preference for each IPI value was measured. To artificially synthesize courtship songs, we used the R libraries *sound* (Heymann 2007) and *Seewave* (Sueur et al. 2008) in R 2.12.2 (R Development Core Team 2013). The four synthesized songs represented a range of IPI from very short to very long (Fig.1; Table1). Artificial song parameter values were determined from previous courtship song recordings of the eight selection lines and of a *D. persimilis* population (14011–0111.49, San Diego). IPI was manipulated, but all other song parameters (interburst interval, number of pulses per burst, pulse length, and carrier frequency) were held constant as we found IPI to be the only parameter that consistently changed between treatments (Snook et al. 2005; Debelle 2013). To reflect the extent of evolutionary divergence in IPI between sexual selection treatments, we used the average IPI values of the replicate showing the largest difference between average E and average M IPI to create an “E-like” IPI and an “M-like” IPI song. This difference in IPI values between E-like IPI and M-like IPI was then used to create another artificial song by subtracting this value from the E-like IPI value, representing an exaggeration of E song with an even shorter IPI (“EE-like” IPI) than what was observed as a result of experimental sexual selection (Fig.1 and Table1). Finally, a song representing the IPI of *D. persimilis* was also synthesized (*persimilis*-like IPI song) which has a longer IPI than *D. pseudoobscura* (Noor and Aquadro 1998). Thus, we have four artificial songs in total: an exaggerated IPI (EE-like) song, a short IPI (E-like) song, a long IPI (M-like) song, and an extended IPI (*persimilis*-like) song.

**Table 1:** Parameters of the artificial courtship songs used in the playback experiment, including the interpulse interval (IPI), the carrier frequency (frequency), the pulse length (PL), and the interburt interval (IBI)

Artificial song	IPI (ms)	Frequency (Hz)	PL (ms)	IBI (s)	Number of pulses
EE-like IPI	33	244.25	12.25	2	30
E-like IPI	36	244.25	12.25	2	30
M-like IPI	39.5	244.25	12.25	2	30
*persimilis*-Like IPI	52	244.25	12.25	2	30

### PLAYBACK DESIGN

To measure female mating preference via mating latency and mating probability, we used playback experiments. Our playback design consisted of three mesh floor chambers (45 × 14 mm) on top of a Torque T70CPA-M speaker, in a darkroom with artificial light, and set on an antivibration table. Artificial songs were played using a Macbook Pro A1286, at a sound intensity level of 85dB (measured using a Radioshack sound level meter 33–2055), corresponding to the particle velocity generated by real courtship songs (Bennet-Clark 1971). Females (*n* = 10) of the same population and replicate (e.g., M3 or E2, etc.) were loaded into each chamber. Females were prestimulated with the artificial courtship song for 2 min (Bennet-Clark et al. 1973; Hall 1986), to additionally minimize any effect of wing clipping on male courtship song. Clipped A males (*n* = 10) were then loaded in each chamber, and the artificial song played for 20 min. Given that courtship and copulation take on average less than 10 min in virgins of this species (Bacigalupe et al. 2007, 2008), and that mated males court and mate sequentially with virgin females within minutes (Crudgington et al. 2009), all females had therefore the opportunity to be courted and to mate during the trial. In addition, selection line females do not re-mate before at least 48 h after the previous mating (Crudgington et al. 2005). Thus, the upper limit for the number of matings per chamber was fixed to a maximum of 10. Each experiment was videotaped (Sanyo VCC-6585P camera, Multicare Electronics Ltd, Leeds, U.K.) and subsequent analysis extracted the number of matings that occurred in each chamber, as well as the mating latency of each mating (defined as the difference between the time of male loading until the start of mating), to estimate female mating preference for IPI. Thirty females were tested per population (nine populations in total) per artificial song (four songs in total), for a total of 1080 females tested, and each female was only tested once.

### PREDICTIONS AND STATISTICS

If female preference covaries with male song, then M-like IPI should be associated with short mating latencies and high mating probabilities for M females (but not for E females), and E-like IPI should be associated with short mating latencies and high mating probabilities for E females (but not for M females). To test these predictions, we analyzed individual female mating probability using a logistic regression with binomial error distribution, and individual mating latency using a mixed model with Gaussian error distribution. In both mixed models, we included female treatment (“E,” “M,” or ancestral “A”; “A” was used as the reference level), artificial song (“EE-like IPI,” “E-like IPI,” “M-like IPI,” and “*persimilis*-like IPI”; “EE-like IPI” was used as the reference level), and female treatment × artificial song interaction as fixed effects. Female replicate (“M1,” “M2,” “M3,” “M4,” “E1,” “E2,” “E3,” “E4,” or “A”) was nested into sexual selection treatment. Playback session (each set of three chambers) was included as random effect. The analysis of mating latency was restricted to the first 50% of the matings occurring in a chamber to minimize the changes in mating conditions (the number of available males nonengaged in mating and the number of virgin females remaining) that could affect the mating latency values of the following matings (Gilbert and Starmer 1985; Casares et al. 1998). For both mating probability and mating latency, estimates of the models were used to represent the average female preference function of each female type (A, E, or M) for each artificial song. Ninety-five percent confidence intervals were also estimated and represented. Normality and homoscedasticity of the residuals were checked graphically. All models were analyzed using the library *lme4* (Bates and Sarkar 2007) in R 2.12.2 (R Development Core Team 2013).

## Results

**Figure 2:**
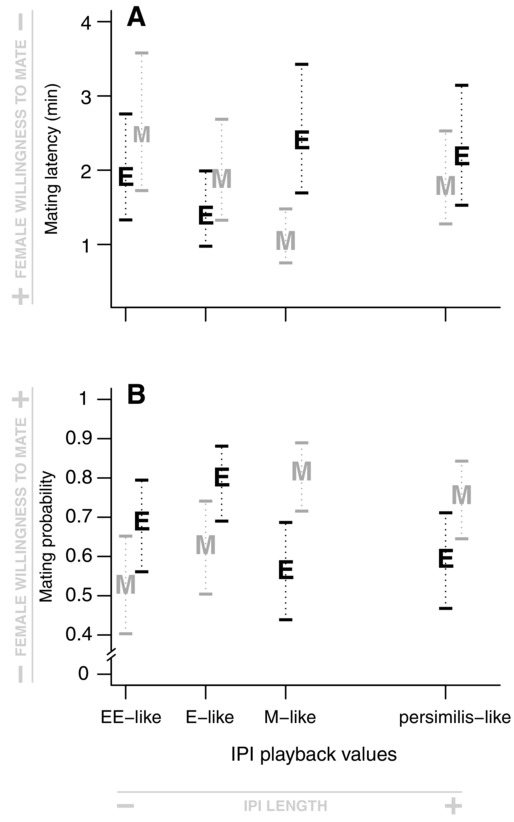
Mating preference functions for IPI of selection line females (E and M) for mating latency (A) and mating probability (B). The two figures show that E and M females present opposite mating preference functions for IPI, for both mating latency and mating probability. The letters represent the fitted values estimated by the mixed-model associated with the four artificial songs, depending on female sexual selection treatment. M is for females from monogamous lines and E is for females from polyandrous lines. Ninety-five percent confidence intervals around each estimated value are represented.

As predicted if sexual selection results in divergent female mating preference for male mating signal, both female mating latency and mating probability are strongly affected by the interaction between female sexual selection treatment and artificial song (Table2). Selection line females show distinct and opposite patterns of mating preference (Fig.2). Females from E lines mate faster under conditions of short IPI values (E-like and EE-like IPIs) and females from M lines mate faster under conditions of long IPI values (M-like and *persimilis*-like IPIs; Fig.2a and Table2). However, there are broadly overlapping confidence intervals, with the exception of preference for M-like IPI. Mating probability is also affected by the interaction between female treatment and artificial song but shows a more pronounced pattern of response compared to mating latency (Fig.2b and Table2). Females from E lines are more likely to mate than females from M lines (80.5% of E females vs. 62.9% of M females mated) when an E-like IPI is being played, whereas females from M lines are more likely to mate than females from E lines (81.3% of M females vs. 56.8% of E females mated) when an M-like IPI is played (Fig.2b and Table2). Further, females from E lines are more likely to mate than females from M lines (68.7% of E females vs. 53.6% of M females mated) with very short IPI values (EE-like IPI), and females from M lines are more likely to mate than females from E lines (76.3% of M females vs. 59.7% of E females) for very long IPI values (*persimilis*-like IPI). Hence, these differences between selection line females extend beyond the limits of experimentally evolved E and M IPI variation.

**Table 2:** Output of the mixed models for mating latency and mating probability analyses, including model estimates and tests statistics

		*Mating latency*			*Mating probability*		
Fixed effects	Factor level	β	LR	*P*	β	LR	*P*
Treatment	M	0.60	3.9	0.14	−1.51	2.7	0.25
	E	0.34			−0.82		
IPI	E-like IPI	−1.0	4.5	0.21	−0.48	2.3	0.51
	M-like IPI	0.13			0.024		
	*persimilis*-Like IPI	−0.13			−0.46		
Treatment × IPI	M- × E-like IPI	0.76	31.48	**2E–05**	0.89	35.7	**3.1E–06**
	E- × E-like IPI	0.73			1.074		
	M- × M-like IPI	−1.024			1.37		
	E- × M-like IPI	0.0842			−0.56		
	M- × *persimilis*-like IPI	−0.19			1.48		
	E- × *persimilis*-like IPI	0.26			0.042		
Global intercept		0.31			1.62		
Random effect variance	Replicate	0.032			0.082		
	Session	0.071			0.097		

The effects of sexual selection treatment, IPI, and their interaction on the response variable (mating latency or mating probability) were tested using likelihood ratio tests. Treatment is the sexual selection treatment (E is for polyandrous and M for monogamous), IPI is the artificial song tested (EE-like, E-like, M-like, or *persimilis*-like IPI), β is the model estimate, LR is the likelihood ratio statistics, and *P* is the *P*-value with bolded values being significant.

**Figure 3:**
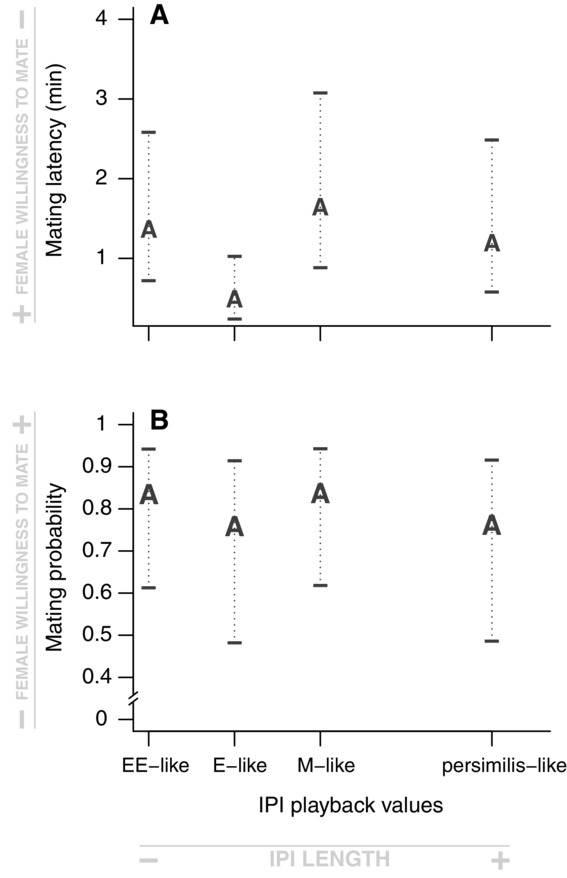
Mating preference functions for IPI of ancestral females, for mating latency (A) and mating probability (B). The two figures show that ancestral females present a relatively flat mating preference function for IPI, for both mating latency and mating probability. The letters represent the fitted values estimated by the mixed-model associated with the four artificial songs. A is for ancestral females. Ninety-five percent confidence intervals around each estimated value are represented.

The confidence interval of ancestral female mating latency (Fig.3a) is wide for both songs with longer IPI values (M-like IPI and *persimilis*-like IPI), but more restricted for the two shorter IPI values (EE-like IPI and E-like IPI). Ancestral females also show a high level of acceptance for all the four IPI values, overlapping either with M or E females ranges, and with greater variability (Fig.3b)

## Discussion

Sexual selection has been suggested to be able to drive mating trait divergence between populations. Yet, direct empirical evidence for this ability is lacking. We used experimental evolution to study the divergence of female mating preference for IPI, a key song parameter for male mating success in *D. pseudoobscura*. IPI diverged in our populations in response to the manipulation of the opportunity for sexual selection, with polyandrous males singing a shorter IPI than monogamous males (Snook et al. 2005; Debelle 2013). Here, we tested for differences in female mating preference for IPI between sexual selection treatments. We show that differences in male courtship song rate can change female mating behavior. Females from polyandrous lines (E) were more likely to mate when presented with a polyandrous-like IPI (E-like IPI), whereas females from monogamous lines (M) were more likely to mate when presented with a monogamous-like IPI (M-like IPI). Mating latency was affected in the same direction as mating probability, although to a lesser degree. Thus, our results suggest that female mating preference has coevolved with male courtship song and diverged between our populations, providing evidence for the divergence of a mating preference for a mating signal, within species, caused by sexual selection manipulation.

Mating trait divergence is generally expected to arise among populations experiencing high sexual selection intensity (West-Eberhard 1983; Panhuis et al. 2001; Ritchie 2007), initiated either by drift (Lande 1981; Uyeda et al. 2009) or differences in environmental conditions (van Doorn et al. 2009), Here, however, we observe that mating trait divergence arose between populations evolving under different sexual selection intensities. Natural populations are often subjected to different ecological and demographic conditions, resulting in different intensities of sexual selection among populations (Lott 1991). Differences in parental investment (Trivers 1972; Emlen and Oring 1977), cost of breeding (Letters et al. 2001), resource distribution (Travis et al. 1995; Streatfeild et al. 2011), operational sex ratio (Weir et al. 2011), or the ability of the environment to propagate signals (Boughman 2001), can result in variation of sexual selection intensity among populations (Arnqvist 1992; Kwiatkowski and Sullivan 2002; Mobley and Jones 2007). Changes in the strength of sexual selection could not only have consequences on the nature of the mating traits targeted by selection, but also on the nature of the sexual selection mechanisms acting on these traits (i.e., direct or indirect selection) and on the form of selection operating. Variation in sexual selection intensity among populations may thus affect both the direction and strength of selection on mating trait in these populations, providing additional opportunities for mating trait divergence to occur.

Mating trait evolution can be fueled by either direct or indirect selection (Andersson 1996; Jennions and Petrie 2000; Kokko et al. 2003, 2006; Jones and Ratterman 2009; Kuijper et al. 2011). In *D. pseudoobscura*, any direct benefits resulting from mate choice are unknown. In the absence of direct benefits and strong direct selection on mating preference, indirect benefits may be sufficient to maintain a mating preference and drive the coevolution of mating traits (Kirkpatrick 1996). E IPI has evolved away from M- and ancestral IPI toward a shorter pulse repetition rate. As pulse repetition rate increases, the cost of this trait is likely to increase. Moreover, short IPI values are associated with an increased overall courtship performance (faster speed to initiate courtship; Snook et al. 2005 and more bursts produced; Debelle 2013). One hypothesis to explain our results is that sexual selection results in directional selection on male courtship song toward short IPI values which may select for more active males (i.e., exhibiting an increased courtship performance) which females from E lines prefer. Under high levels of male–male competition, selecting more active males could constitute a substantial source of indirect benefits via good genes or sexy sons (Wong and Candolin 2005). How females gain from their mate preference and the costs of such choice has yet to be studied.

Females from M lines evolved under enforced monogamy, and yet discriminate against E-like values of IPI. Female preference is expected to be strongly counterselected when its expression results in direct costs for the female (Jennions and Petrie 1997). Fertility costs can be very important for females in the monogamous lines, as refusing to mate with the one randomly assigned male in her vial will result in zero fitness. However, a preference can be maintained when the benefits of expressing it outweigh the costs (Jennions and Petrie 1997). Contrary to females from E lines, females from M lines mate preferentially with a long IPI, and therefore may benefit from a selective advantage by favoring males who invest less in courting. Direct costs of male–female sexual interactions have been investigated in our selection lines, as well as other *Drosophila* species. Male courtship inflicts longevity costs to both males and females (Partridge and Farquhar 1981; Partridge et al. 1987; Partridge and Fowler 1990; Cordts and Partridge 1996; Holland and Rice 1999; Friberg and Arnqvist 2003). Female fecundity is negatively affected by male courtship intensity in *D. melanogaster* (Friberg and Arnqvist 2003), and by male-biased sex ratios in *D. pseudoobscura* (Crudgington et al. 2005). Males and females share similar reproductive interests in M populations, and therefore reducing direct costs due to courtship and matings is likely to be under strong direct selection. The long IPI values of males from M lines, preferred by females from M lines, may be associated with less intense courtship and less costly matings. Indeed, males from M lines court less and achieve fewer matings than males from E lines (Crudgington et al. 2009), therefore reducing the direct courtship and mating costs on both females and males. A relaxation of sexual conflict may thus have driven the evolution of M female preference in the opposite direction of E female preference, as accepting to mate with less-active and less-harmful males is likely to be selected for in a monogamous context. Hence, mating trait coevolution may likely be resulting from the action of two different selection mechanisms, causing the mating traits of the two sexual selection treatments to evolve in different directions. More work will be needed to identify the exact selective mechanisms involved in the observed divergence of mating traits.

Population-level variation in mating preference is required for mating trait divergence to evolve, but remains relatively poorly studied (Jennions and Petrie 1997; Wagner 1998; Widemo and Sæther 1999; Bailey 2008). Here, we observe an absence of discrimination against IPI by ancestral females at the population level, whereas selection line females actively discriminate between different IPI values. As IPI has been previously shown to influence *D. pseudoobscura*–*persimilis* hybrid male mating success (Williams et al. 2001), a discrimination in our ancestral population against *persimilis*-like IPI could be expected. However, as the geographical location of the ancestral population used in this study was both outside the range of *D. persimilis* distribution and different from the population used by Williams et al. (2001), discrimination against *persimilis*-like IPI in our ancestral population is a necessary outcome. Furthermore, the nondiscriminatory pattern of ancestral females against the entire range of IPI values at the population level does not mean an absence of discrimination against IPI at the individual level in this population (Wagner 1998; Widemo and Sæther 1999). The fact that IPI values are differentially discriminated against in the selection lines derived from the ancestral population, and this in opposite directions between the sexual selection treatments, suggests that variation in mating preference for IPI is present in the ancestral population (or has been present at first and secondarily been lost; Wiens 2001). The flat mating preference function observed at the population level in the ancestral population may thus reflect the variation in mating preference present in this population (both at the intra- and interindividual levels) recruited in contrasting directions by sexual selection in the selection lines. Investigating the structure of mating preference variation is essential to understand and predict the divergence of mating traits between populations, and ultimately species (Maan and Seehausen 2011; Weissing et al. 2011), and thus studying mating preference variation in our populations may help to determine the impact of variation structure on the possible direction of mating trait evolution via sexual selection (Jennions and Petrie 1997; Wagner 1998).

In conclusion, our results suggest that female mating preference for IPI coevolved with male IPI divergence in our populations. Manipulating the opportunity for sexual selection has resulted in the divergence of mating traits between populations, providing direct evidence supporting the ability of sexual selection to drive mating trait coevolution. The next step is to test if this divergence in mating traits generates sexual isolation between populations experiencing different intensities of sexual selection, to understand whether sexual selection has the potential to be an important driving force in speciation.

## DATA ARCHIVING

The doi for our data is doi:10.5061/dryad.3h7g8.

## Supporting Information

Figure S1 Wing clipping performed on the ancestral males used in the playback experiment.
