# Supplementary material for: EVOLUTION OF DIVERGENT FEMALE MATING PREFERENCE IN RESPONSE TO EXPERIMENTAL SEXUAL SELECTION
Source: Evolution. 2014 Jul 21;68(9):2524–33. doi: 10.1111/evo.12473 (PMC4262321; doi:10.1111/evo.12473)
Supplement: Figure S1 — Wing clipping performed on the ancestral males used in the playback experiment. [file evo0068-2524-SD1.pdf]

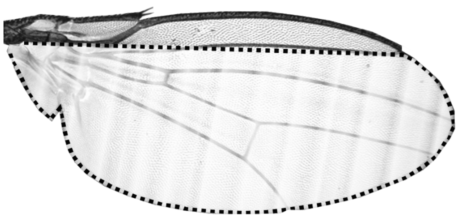

Figure S1. Wing clipping performed on the ancestral males used in the playback experiment.

Both wings were clipped (the zone whitened and circled by a dashed line was removed).
